# PERMMA: Enhancing parameter estimation of software reliability growth models: A comparative analysis of metaheuristic optimization algorithms

**DOI:** 10.1371/journal.pone.0304055

**Published:** 2024-09-04

**Authors:** Vishal Pradhan, Arijit Patra, Ankush Jain, Garima Jain, Ajay Kumar, Joydip Dhar, Anjan Bandyopadhyay, Saurav Mallik, Naim Ahmad, Ahmed Said Badawy

**Affiliations:** 1 School of Applied Sciences, Kalinga Institute of Industrial Technology, Odisha, India; 2 Department of Computer Science & Engineering, Netaji Subhas University of Technology, Dwarka, New Delhi, India; 3 Department of Computer Science and Business System, Noida Institute of Engineering and Technology, Greater Noida, India; 4 Department of Engineering Sciences, ABV-Indian Institute of Information Technology and Management Gwalior, Gwalior, MP, India; 5 School of Computer Science and Engineering, Kalinga Institute of Industrial Technology, Odisha, India; 6 Department of Environmental Health, Harvard T H Chan School of Public Health, Boston, MA, United States of America; 7 Department of Pharmacology & Toxicology, University of Arizona, Tucson, AZ, United States of America; 8 College of Computer Science, King Khalid University, Abha, Saudi Arabia; SR University, INDIA

## Abstract

Software reliability growth models (SRGMs) are universally admitted and employed for reliability assessment. The process of software reliability analysis is separated into two components. The first component is model construction, and the second is parameter estimation. This study concentrates on the second segment parameter estimation. The past few decades of literature observance say that the parameter estimation was typically done by either maximum likelihood estimation (MLE) or least squares estimation (LSE). Increasing attention has been noted in stochastic optimization methods in the previous couple of decades. There are various limitations in the traditional optimization criteria; to overcome these obstacles metaheuristic optimization algorithms are used. Therefore, it requires a method of search space and local optima avoidance. To analyze the applicability of various developed meta-heuristic algorithms in SRGMs parameter estimation. The proposed approach compares the meta-heuristic methods for parameter estimation by various criteria. For parameter estimation, this study uses four meta-heuristics algorithms: Grey-Wolf Optimizer (GWO), Regenerative Genetic Algorithm (RGA), Sine-Cosine Algorithm (SCA), and Gravitational Search Algorithm (GSA). Four popular SRGMs did the comparative analysis of the parameter estimation power of these four algorithms on three actual-failure datasets. The estimated value of parameters through meta-heuristic algorithms are approximately near the LSE method values. The results show that RGA and GWO are better on a variety of real-world failure data, and they have excellent parameter estimation potential. Based on the convergence and *R*^2^ distribution criteria, this study suggests that RGA and GWO are more appropriate for the parameter estimation of SRGMs. RGA could locate the optimal solution more correctly and faster than GWO and other optimization techniques.

## 1 Introduction

Nowadays, the requirement for software systems has been overgrown because of its extensive application-based area; because of the revved technical advancement, the demand for multi-purpose software systems increases. In versatile software development, the size and complexity of software increases, so producing high-quality software becomes a more crucial task for software developers. The enormous complexity process of the software development makes it complicated for software developers to build high-grade software. The software has become an integral part of industry and modern society, e.g., nuclear reactor, defense, patient health monitoring, industrial process, banking, transportation, telecommunications, personal entertainment, home appliances, etc. [1, 2].

The software is a program in the form of a set of instructions or a line of codes. In the twenty-first era, all corporations or even individual personalities depend on software-based systems. Now, almost everyone is associated with computer systems, either partially or entirely. Software defects have emerged in disastrous failures, with few with terrible outcomes. There are few cases of devastating defeats solely due to software crashes. Newly, many banks and digital banking institutions felt software crashes in the form of misplaced data or information is a jumble. Software industries must assess their reliability when releasing software products in the market. Reliability prediction of software becomes the most prominent measure to avoid software failure and its maintenance expenses. User feedback generally indicates reliability; however, this method does not help evaluate software reliability before release. However, software reliability growth models (SRGMs) serve to present this information before release. There are various features for reckoning software quality; software reliability is a comprehensively utilized metric for software excellence quality [3].

The operating probability, or the probability of completing the expected function between the assigned scenario of the environment over a specified period with defined, designed, and restricted circumstances, is directed to software reliability [4]. We need a sophisticated way to formulate SRGMs to quantitatively estimate software reliability [5]. SRGMs try to correlate the failure data with distinguished distributions such as exponential, Weibull, logistic, etc. Over the previous few decades, diverse growth models have been ormulated for reliability growth estimation and software failure prediction [6–10]. Several pieces of research on software failure forecasts have just obtained considerable engagement since they helped the testing group [11]. Many environmental elements are considered in the existing literature to enhance the accuracy of the SRGMs. Recentally, Kim et al. [12] proposed a new SRGM and optimal release time with dependent failure. In this work, they derive the SRGM and assuming point symmetry fault detection rate.

A large number of growth models based on the non-homogeneous Poisson process (NHPP) have been created in the literature to measure software reliability in a realistic environment [13, 14]. Because of their ubiquity and performance in practice, NHPP-SRGMs are the most used type of growth model to estimate reliability [15–17]. The NHPP is a counting procedure, and the SRGMs based on it predict the amount of identified problems over time. The Goel and Okumoto (GO) model is one of the most well-known and early NHPP-based SRGMs [18]. In order to build realistic SRGMs, these studies contain several assumptions in their models. These growth models also assist in operational phase decision-making for the software’s to ensure required reliability [19]. In previous studies, the majority of growth models considered the traditional hypothesis, i.e., perfect debugging, while only a small amount of research has been done on an imperfect debugging environment [20–22].

Failure data estimate the parameters of these models through several estimation methods [23]. After the completion of parameter estimation, the SRGMs can be utilized to analyze several performance criteria. For the stochastic system, the parameter estimation problem can be transformed into an issue of optimization. The purpose of estimation methods is to know a collection of parameters that are the most desirable parameters to fit the function and outline the failure data perfectly. There are various analytical/ numerical methods present to find the value of unknown parameters such as maximum likelihood estimation (MLE) and least squares estimation (LSE), etc. This work considers two, three, four, and five parameters existing concave and S-shaped SRGMs, i.e., GO, Inflection S-shaped model (ISS), PNZ, and PZM models, respectively [1]. These model parameters are estimated by numerical (LSE) and various meta-heuristic optimization techniques, i.e., RGA, GSA, SCA, and GWO. The ultimate objective of the suggested work is to find a more satisfying parameter estimation accuracy for the growth models. Existing SRGMs call for numerical approaches, MLE, or LSE to estimate parameters. However, these methods put significant limits on SRGM parameter estimation, such as requiring the modelling function’s continuity and the existence of derivatives.

The original assistance of this assignment is outlined as follows:

1. To analyze the applicability of various developed meta-heuristic optimization techniques in the area of SRGMs parameter estimation.
2. To make the comparative analysis of various meta-heuristic optimization techniques for the optimal parameter estimation and identify the most appropriate algorithms for the same.

This work conduct the comparative analysis for four well-established SRGMs, GO, ISS, PNZ, and PZM model on three real-failure datasets. The leftovers of this article are systematized as follows. Section 2 confers the related literature. NHPP and NHPP based SRGMs are debated in Section 3. Parameter estimation techniques MLE, LSE and meta-heuristic optimization techniques are represented in Section 4. Section 5 discusses the proposed methodology. Section 6 presents the evaluation process and experiment report based on the numerical investigation of data sets. Finally, Section 7 completes the assignment with forthcoming suggestions.

## 2 Literature survey

This section delivers detailed literature related to the SRGM models and their respective parameter estimation techniques. In 2018, [24] consider the testing coverage as well as the operational environment’s uncertainty or randomness. They established a novel NHPP-based software reliability model using testing coverage with an uncertain operating environment. In this research, they show a sensitivity analysis to investigate the influence of individual parameters of the presented model. They use the LSE method to estimate the reliability model’s parameter. In 2019, the reliability evaluation of software based on a NHPP introduces a generalized model, [25] included the unpredictability of the operational conditions and its impact on the rate of defect detection to encompass imprecise debugging. This article uses a general model to construct operational environment uncertainty models, allowing for flexibility in incorporating a different random environmental element and fault detection rate. MLE may be unable to get precise estimates in some cases, mainly when u(t) is too intricate, and we must resort to LSE. As a result, they employ the LSE approach for parameter estimation.

In 2020 under the martingale framework, [26] suggested a generalized numerous environmental aspects based SRGM and associated unpredictability. The unpredictability is reflected in the software defect detection mechanism; this is indeed a stochastic defect detection procedure. For example, a multi-environmental-variables SRGM is further refined, integrating two unique environmental aspects, the portion of reused modules and the frequency of programme specification modification. They employ LSE to estimate model parameters.

The literature demonstrates the limitations of the numerical estimate, such as the fact that it is rarely trivial. For small samples, the estimate can sometimes reveal significant biases. The first parameter value selection is likewise a delicate matter. Some academics presented a neural network established strategy for outlining the software failures non-linearity [27–30]. The foremost downside of these methods is that they need an extensive training dataset to teach the technique, which results in a significant increase in computing cost and time. These approaches also incur significant computational expenses in order to forecast the number of failures per iteration. Nature-inspired solutions are adopted in numerous software testing, software reliability, and software engineering disciplines to solve the aforementioned restriction [31]. Meta-heuristics are straightforward ideas primarily inspire from animal manners, biological circumstances, and evolutionary conceptions are typical seeds of motivation. Recently, Various algorithm are are developed for various optimization problems [32–38]

For parameters optimization of SRGMs, the intelligent algorithm of optimization is used. Intelligent optimization methods do not require any additional hypotheses. In 1995, [39] presented a prototype for SRGMs using GA and discovered a different steady way of obtaining an estimation three decades ago. In 2013, [40] suggested a renewed estimation technique for SRGM, namely the PSO methodology, albeit it should be noted that this method required a broad search range and a slow convergence rate. In 2009, [41] recommended that for SRGM, a multi-objective GA be used. In 2010, To enhance the implementation of essential GA for directing the estimating concern of reliability models, [42] suggested a modified GA (MGA) based estimation approach.

In 2011, [43] studied a parameter estimate technique established on the Ant-Colony-Algorithm. Three collections of actual defects datasets are used to generate numerical examples presented and explored in depth. It is demonstrated that (1) the standard technique fails to find feasible explanations for a few datasets and SRGMs, whereas the suggested technique always does; (2) when compared to the PSO, various technique’s outcomes are roughly ten times more accurate for most models. Several techniques exceed the PSO algorithm in representations of convergence rate and precision. Our future research will focus on the initial value setting of parameters as well as the way of splitting solution space. In 2013, [44] proposes that the SRGM parameter estimation problem be solved using the PSO algorithm and then compares the results to those obtained using GA. Data from 16 other projects back up the findings. The PSO results show a high predictive capacity, as evidenced by the minimal fallacy forecasts. The outcomes acquired by PSO are superior to those accepted by GA. As a consequence, PSO can be utilized for SRGM parameter estimation, and the findings were validated using 16 projects. They analyzed the data and compared it to the GA approach.

In 2015, [45] performed a comparative analysis between CGA, BGA, and RGA. They used five typical SRGMs to conduct tests on eight failure datasets. The researchers used real-valued GA for parameter estimation and concluded that the optimal solution could be located more correctly by RGA and faster than previous GA techniques. They propose in their future work to do a comperative analysis of more meta-heuristics in SRGMs parameter estimation. We plan to execute a relatively practical investigation of RGA and other techniques for SRGMs parameter estimation in the future. To optimize these parameters of TE based SRGM in 2016 [46] investigates the application and advancement of a swarm intelligent system, specifically the quantum particle technique. The suggested SRGM-TEF model’s performance with improved parameters is analogized to different current models. The investigation findings indicated that the suggested parameter estimation strategy utilizing quantum particles is advantageous and versatile. One can attain better reliability performance by employing SRGM-TEF on various software failure datasets.

The results of [47] show that a technique based on a GSA for estimating parameters solves these issues and provides outstanding grade parameter estimation. Comprehensive experiments on nine real datasets were carried out in this research, with the results assessed to compare the suggested method. The investigation results show that the suggested strategy outperforms existing estimation, GA, and cuckoo search methods. This research discusses an effective parameter estimate strategy for SRGMs using GSA that overcomes the constraints of prior approaches. Ample investigations on several popular datasets for various notable SRGMs are used to evaluate the suggested technique. In 2018, [48] proposed a multi-release fault dependency SRGM for open-source software. In this paper, they employed a GA algorithm for solving the optimization function.

## 3 Software reliability growth models

### 3.1 Non-homogeneous poisson process

In the computing procedure, {ℵ(*t*), *t* ≥ 0} stands for the total numeral of diagnosed defects, while NHPP stands for time-dependent failure intensity ℏ(*t*), i.e.,

$$\begin{array}{c} \vartheta \left(t\right)=\int_{0}^{t}\hbar \left(t\right)dt. \end{array}$$ (1)

Here, *ϑ*(*t*) is the expected absolute amount of faults present in the system by time *t*, i.e., mean value function (MVF). So, the probability that the *κ* defects appears by time *t* is,

$$\begin{array}{c} P\left\{ℵ\left(t\right)=\kappa \right\}=\frac{{\left(\vartheta \left(t\right)\right)}^{\kappa }e^{-\vartheta (t)}}{\kappa !},\;\kappa =0,1,2\ldots \end{array}$$ (2)

### 3.2 NHPP based SRGMs

There are four existing models considered in this study. Out of four, the first two models are developed in perfect debugging(PD), and the other two are imperfect debugging(ID) phenomena. In ID phenomenon, the defect scope function is an exponential and linear time-dependent function. For estimation evaluation criteria, the selected four SRGMs are explained below:

1. **GO model** [18]
  The typical hypothesis for all NHPP models is one of the G-O model’s assumptions. The expected amount of noticed defects in (*t*, *t* + Δ*t*) is proportionate to the amount of defects staying in the system. Thus, the MVF of the G-O model can be depicted as:
  $$\begin{array}{c} \vartheta \left(t\right)=T\left(1-e^{-\mu t}\right). \end{array}$$ (3)
  Here, T is the initial amount of defects to be noticed, and *μ* is the fault identification rate, i.e., *μ*(*t*) is taken as point symmetry. It is a concave type model.
2. **ISS model** [49]
  In this model, the hypothesis is that the identification rate is time-dependent. Detection rate incorporated learning factor *ξ* and nature of detection rate is S-shaped. Therefore, the MVF of the SRGM can be represented as:
  $$\begin{array}{c} \vartheta \left(t\right)=\frac{T(1-e^{-\mu t})}{1+\xi e^{-\mu t}}. \end{array}$$ (4)
  Here, $\mu \left(t\right)=\frac{\mu }{1+\xi e^{-\mu t}}$ and *μ*(*t*) is taken as point symmetry. GO model is special case of ISS model when *μ* = 0. It is also a concave type model.
3. **PNZ model** [50]
  In this model, authors assume that defects may be raised during detection. The fault content rate is linear of testing time, i.e., T(t)=T(1+ζt) and the fault identification rate is a non-decreasing ISS function. Therefore, the MVF of the SRGM is represented as:
  $$\begin{array}{c} u\left(t\right)=\frac{T([1-e^{-\mu t}][1-\frac{\zeta }{\mu }]+\zeta Tt)}{1+\xi e^{-\mu t}}. \end{array}$$ (5)
  Here, $\mu \left(t\right)=\frac{\mu }{1+\xi e^{-\mu t}}$, *μ*(*t*) is taken as point symmetry and *ξ* is leaning factor. When *ζ* = 0, PNZ model becomes ISS model and when *ζ* = 0 and *ξ* = 0, PNZ model becomes a GO model. It is S-shaped and concave type model.
4. **PZM model** [1]
  In this model, authors assume that the defects may be raised during the detection. The fault content rate is exponential function of testing time, i.e., $T\left(t\right)=\varsigma +T\left(1-e^{-\mu t}\right)$ and the fault detection rate is same as PNZ model. Therefore, the MVF of the SRGM is represented as:
  $$\begin{array}{c} \vartheta \left(t\right)=\frac{\left(\left(\kappa +T\right)[1-e^{-\mu t}])-[(\frac{T}{\mu -\zeta })(e^{-\zeta t}-e^{-\mu t})\right]}{1+\xi e^{-\mu t}}. \end{array}$$ (6)
  Here, $\mu \left(t\right)=\frac{\mu }{1+\xi e^{-\mu t}}$ and i.e., *μ*(*t*) is taken as point symmetry. When *ζ* = *μ* and *κ* = 0, PZM model become a ISS model and when *ζ* = *μ*, *κ* = 0 and *ξ* = 0, PZM model become a GO model. This SRGM is also says generalized NHPP SRGM. It is also a S-shaped and concave type model.

The brief detail of all the SRGMs are given in Table 1, which is considered for comparative analysis.

**Table 1:** Software reliability growth models for algorithm evaluation.

*SRGMs*	Model type	MVF (*ϑ(t)*)
*G-O model*[18]	Concave (C)	$T(1-e^{-\mu t})$
*ISS model* [49]	Concave (C)	$\frac{T(1-e^{-\mu t})}{1+\xi e^{-\mu t}}$
*PNZ model* [50]	S-shaped and concave (S&C)	$\frac{T([1-e^{-\mu t}][1-\frac{\zeta }{\mu }]+\zeta Tt)}{1+\xi e^{-\mu t}}$
*PZ model* [1]	S-shaped and concave (S&C)	$\frac{\left(\left(k+T\right)[1-e^{-\mu t}])-[(\frac{T}{\mu -\zeta })(e^{-\zeta t}-e^{-\mu t})\right]}{1+\xi e^{-\mu t}}$

After development of SRGM, the succeeding step is parameter estimation. Here, the parameter estimation is classified into two categories based on the estimation techniques: (i) traditional estimation techniques, (ii) meta-heuristic optimization techniques.

## 4 Parameter estimation techniques

### 4.1 Numerical approaches

There are two most used parameter estimation techniques in literature, i.e., LSE and MSE, and in between them, most of the study uses LSE.

#### 4.1.1 Maximum likelihood estimation (MLE)

MLE is one of the most effective methods for calculating estimators. In contrast to other estimating approaches, MLE are asymptotically normal and consistent and as the size of sample increases. For statistical models, MLE is a robust parameter estimation method. MLE has various meaningful statistical attributes of the optimal estimator for a significant quantity of data. The explication method of evaluation of parameters through MLE is very complicated. When the failure data meets certain hypothesis, like as a specified distribution, MLE can be employed. They require a numerical solution [41, 42, 51], which is a reasonable obstacle for the management team. The log-likelihood functions of SRGM are a little complex, etc., which further creates MLE quite complicated. In recent literature, various researchers used LSE for parameter estimation.

Let Π_*i*_ is cumulative number of detected defects up to *t*_*i*_, *i* = 1, 2, …,

$$\begin{array}{c} L=\prod_{i=1}^{n}[\frac{{\left[\vartheta \left(t_{i}\right)-\vartheta \left(t_{i-1}\right)\right]}^{\Pi_{i}-\Pi_{i-1}}}{(\Pi_{i}-\Pi_{i-1})!}]e^{-(\vartheta \left(t_{i}\right)-\vartheta \left(t_{i-1}\right))},\\ \text{log}\left(L\right)=\sum_{i=1}^{n}\left[\left(\Pi_{i}\;-\;\Pi_{i-1}\right)\cdot \text{log}\left(\vartheta \left(t_{i}\right)\;-\;\vartheta \left(t_{i-1}\right)\right)-\left(\vartheta \left(t_{i}\right)\;-\;\vartheta \left(t_{i-1}\right)\right)\;-\;\text{log}\left[\left(\Pi_{i}\;-\;\Pi_{i-1}\right)\right]\right]. \end{array}$$ (7)

Carry the derivative on both sides concerning parameters which are unknown and put zero after obtaining the individual likelihood function; then, the system of equations is solved to get the value of parameters that are unknown. However, MLE may not be able to obtain accurate estimates in some cases, particularly when u(t) is too intricate, and we must resort to LSE. As a result, we’ll talk about how the model parameters are assessed by employing the LSE approach.

#### 4.1.2 Least square estimation (LSE)

LSE process identifies the most likely accurate set of parameters for an assigned experimental dataset [42]. Besides, it practices a curve fitting method on the test dataset for unknown parameter estimation [39]. LSE is straightforward to use, and most of the software has non-linear regression functionality as a tool [45]. It displays the selection, while MLE cannot produce a pleasant vale of the estimated parameter. It suggests steady results in more comprehensive datasets; therefore, it reveals a notably adopted technique adopted by the practitioners in software industries. The previous work reveals the flaws in numerical estimate methodologies, such as being frequently non-trivial. For limited samples, the estimate procedure usually exposes significant biases. Another delicate issue is determining the parameter’s initial value. The regression analysis and estimation of parameter can be done using a variety of statistical approaches, including standard LSE, non-linear LSE (N-LSE), and MLE [52]. The N-LSE procedure, out of the three, confesses sampling error and all additional flaws in the estimation of parameter, and so outperforms the other two methods for the validation of model [53]. The NLS has been carried out using The N-LSE was performed using Levenberg-approach, Marquardt’s, which reduces the total sum of squared errors [54, 55].

Allow all failure data to be expressed in couples (*t*_*j*_, Π_*j*_) $\left(j=1,2,\ldots ,z;0<t_{1}<t_{2}<\ldots <t_{z}\right)$, where Π_*j*_ is the total amount of defects noticed over time (0, *t*_*j*_]. The following is the sum of the squared distances:

$$\begin{array}{c} S=\sum_{j=1}^{z}{\left(\Pi_{j}-\vartheta \left(t_{j}\right)\right)}^{2} \end{array}$$ (8)

We may get equations for the proposed model by carrying derivatives of Eq (8) concerning each SRGM parameter and fixing the outcomes equivalent to zero:

$$\begin{array}{c} \frac{\partial S}{\partial T}=\frac{\partial S}{\partial \mu }=\frac{\partial S}{\partial \xi }=\frac{\partial S}{\partial \zeta }=\frac{\partial S}{\partial k}=\ldots =0 \end{array}$$ (9)

We may get the LSE for SRGMs of all parameters by simultaneously solving the preceding equations.

### 4.2 Meta-heuristic approaches

Heuristics are procedures that explore good (near-optimal) solutions at a moderate cost of computation without ensuring either optimality or feasibility” [56]. Heuristic algorithms mimic biological or physical rules. Several popular algorithms like BFA, PSO, SA, ACO, and genetic algorithm (GA) are used for parameter estimation [57, 58]. Two essential features, exploration and exploitation, are recognized in population-based algorithms. Investigation is the strength to grow search space, while exploitation is the strength to obtain the nearby right solution, i.e, optima. These algorithms must apply the exploration in the initial some iterations to bypass the local optima stagnation. Therefore, exploration is an essential concern in a population-based algorithm [59]. A primary solution is a proper trade-off between exploitation and exploration to achieve an excellent performance [60]. Despite every population-based search algorithms giving competent decisions, no heuristic algorithm could perform better than another in determining all optimizing difficulties. An algorithm may give worse than others in some problems and, in some cases, solve better than others. A hybrid global optimization algorithm is proposed for objective functions typical of non-linear least squares regression problems. It involves three components: simplifying the feasible region, excluding areas near local minimizers, and efficiently finding local minima [61]. In 2010 Žilinskas and Žilinskas explores the use of global optimization algorithms for nonlinear regression problems, with a focus on interval arithmetic-based methods. Unimodality of optimization problems in nonlinear regression is typically uncertain [62].

These algorithms are broadly classified into two categories: population-based versus individual-based algorithms. More than one solutions are created in a population-based optimization method. Throughout the iterations, the set of solutions are improved. However, this method initializes solely a single solution and grows/updates it throughout the iterations. The population-based algorithms serve as an escape from high local optima because they use many solutions. Various answers also help a population-based method to learn from distinct territories of the exploration area quickly. During the optimization process, information interchange is made among the search agents. Hence, search agents can explore and exploit search spaces adequately and faster. The major disadvantage of these techniques is the huge amount of evaluation through various functions.

These methods are free from constraints. In this study, we implemented one swarm intelligence, one evolutionary, and two physics-based algorithms for parameter estimation.

#### 4.2.1 Regenerated GA (RGA)

A basic plan of conventional RGA [63] is as follows: (i) generate a random initial population of chromosomes, (ii) a prescribed fitness function is used to compute chromosomes, (iii) for subsequent generation picked candidates from the population, (iv) implementing the genetic operators of crossover and mutation to this picked sub-population for creating a new population. The following crossover is used for chromosomes with value encoded.

$$\begin{array}{c} offspring_{1}=parent_{1} \end{array}$$ (10)

$$\begin{array}{c} offspring_{2}=parent_{1}\pm r_{c}*\left(parent_{1}-parent_{2}\right) \end{array}$$ (11)

Here, *r*_*c*_ is a random value between 0 and 1. As the generations pass, the range decreases. For mutation operator the equation is as follow:

$$\begin{array}{c} offspring_{i}\left(P_{i}\right)=\{\begin{array}{cc} & parent_{i}+f\left(G\right)*\left(upperboud_{i}-parent_{i}\right)\\ & \;or\\ & parent_{2i}-f\left(G\right)*\left(parent_{i}-lowerboud_{i}\right) \end{array} \end{array}$$ (12)

Here, *f*(*G*) is the range function considering the current generation (G) number, the function is as follows:

$$\begin{array}{c} f\left(G\right)={\left(r_{e}*(1-\frac{G}{G_{max}})\right)}^{b} \end{array}$$ (13)

Here, *G*_*max*_ is the maximum number of generations and b is a shape parameter. Algorithm 1 represents the pseudo-code of RGA. This algorithm showed that the nature-inspired models could be straightforward and effective in optimizing problems.

**Algorithm 1** Pseudo code of RGA.

 **Input:** Number of agents (*NA*), Maximum iterations (*Max*_*iter*), Current iteration (*Iter* = 0), Crossover rate (*C*_*r*_), Mutation rate (*M*_*r*_), Objective function (*f*)

 **Output:** Best agent (*P*_*best*_)

1: **procedure** RGA (*NA*, *Max*_*iter*, *f*, *C*_*r*_, *M*_*r*_)

2:  Initialize the agents (*P*).

3:  Compute the fitness of all agents through function *f*.

4:  Identify the current best parents by top-mate selection (*P*_*best*_).

5:  **while** *Iter* < *Max*_*iter* **do**

6:   **for each** search agent *i* ∈ (*NA*) **do**

   /* Perform Crossover */

7:    *r*_*c*_ = *rand*(0, 1) /* Here, *rand*(*x*, *y*) generates the uniform random number in range [0, 1]. */

8:     **if** *r*_*c*_ < *C*_*r*_ **then**

9:      Randomly pick a agent *P*_*r*_ from the population for crossover operation.

10:     Produce heuristic crossover breed *P*_*c*_ by performing arithmetic crossover using Eqs 10 and 11.

11:    **end if**

     /* Perform Mutation */

12:    *r*_*m*_ = *rand*(0, 1)

13:    **if** *r*_*m*_ < *M*_*r*_ **then**

14:     Generate new solution by non-uniform mutation.

15:     Re-initialize dimension *d* of crossover breed *P*_*c*_.

16:    **end if**

     /* Perform Selection */

17:    **if** *f*(*P*_*c*_)<*f*(*P*_*i*_) **then**

18:     *P*_*i*_ = *P*_*c*_

19:    **end if**

20:   **end for**

21:   Compute the fitness of all search agents through function *f*.

22:   Update *P*_*best*_ agent.

23:   *Iter* = *Iter* + 1

24:  **end while**

25: **return** *P*_*best*_

26: **end procedure**

#### 4.2.2 Gravitational Search Algorithm (GSA)

The GSA is a well-established optimization method based on the mass interactions and law of gravity. In the GSA algorithm, the explorer agents are the set of masses that communicate with each other by the laws of motion and Newtonian gravity [64]. In this, agents are viewed as objects, and masses estimate their performance. All those objects pull all others by gravity, and this force creates a global action of every object in the direction of the objects with more massive masses.

Researchers proposed simply a collection of agents with higher mass employ their force to others. But, remain cautious of applying this method because it may decrease the exploration potential and improve the exploitation capacity. Developer recalls that to bypass fooling in local optimum, the algorithm needs to utilize the exploration at the start. To enhance GSA’s performance by managing exploitation and exploration, only the *K* − *best* agents will pull the others. *K* − *best* is a time-dependent function, with the opening value *K*_0_ at the start and reducing with time. In the starting, all agents employ the force, and as time crosses, *K* − *best* is reduced linearly, and at the finish, one agent employs force to the others.

We define the force acting on mass I from mass ‘j’ at a certain moment ‘t’ as follows:

$$\begin{array}{c} F_{ij}^{d}\left(Iter\right)=G\left(Iter\right)\frac{M_{pi}\left(Iter\right)\times M_{aj}\left(Iter\right)}{R_{ij}\left(Iter\right)+\epsilon }\left(X_{i}^{d}\left(Iter\right)-X_{j}^{d}\left(Iter\right)\right) \end{array}$$ (14)

$$\begin{array}{c} v_{i}^{d}\left(Iter+1\right)=rand_{i}\times v_{i}^{d}\left(Iter\right)+a_{i}^{d}\left(Iter\right) \end{array}$$ (15)

$$\begin{array}{c} x_{i}^{d}\left(Iter+1\right)=x_{i}^{d}\left(Iter\right)+v_{i}^{d}\left(Iter+1\right) \end{array}$$ (16)

Here, the position of the *i*th agent in the *d*th dimension is represented by $x_{i}^{d}$. *a*_*i*_ is the acceleration of the agent*i* at time *t*, and in direction *d*th *rand*_*i*_ is a uniform random variable with a value between 0 and 1. We use this random integer to give the search a randomized characteristic. Pseudo code of GSA is described in algorithm 2.

**Algorithm 2** Pseudo code of GSA.

 **Input:** Number of agents (*NA*), Maximum iterations (*Max*_*iter*), Current iteration (*Iter* = 0), Objective function (*f*)

 **Output:** Best agent (*P*_*best*_)

1: **procedure** GSA (*NA*, *Max*_*iter*, *f*, *Iter* = 0)

2:  Initialize each agent *P*_*i*_. /* Here, *i* ∈ *NA* */

3:  Initialize algorithmic parameters, i.e., gravitational field (*G*(*Iter*)), and Mass (*M*(*Iter*)).

4:  Compute the fitness of all agents through function *f*.

5:  Identify the current best agent (*P*_*best*_) and worst agent (*P*_*worst*_) of the population.

6:  **while** *Iter* < *Max*_*iter* **do**

7:   Calculate the total force in each direction through Eq 14.

8:   Calculate the acceleration and velocity in each direction.

9:   **for each** search agent *i* ∈ (*NA*) **do**

10:    Update the position of agent *P*_*i*_.

11:    Evaluate the fitness of *P*_*i*_ through objective function *f*.

12:   **end for**

13:   Update the *P*_*best*_ and *P*_*worst*_ of population through updated population.

14:   Update algorithmic parameters *G*(*Iter*), *M*(*Iter*).

15:   *Iter* = *Iter* + 1

16:  **end while**

17: **return** *P*_*best*_

18: **end procedure**

#### 4.2.3 SCA: Sine Cosine Algorithm

The SCA algorithm [65] explains that elementary mathematics is applied to compose optimization process. This algorithm is used in various areas [66]. As mentioned earlier, the algorithm introduced the uses of sine and cosine functions to exploit and explore the space among two solutions to obtain a fitter solution in the search space. The SCA produces many initial random solutions and needs them to undulate towards or outwards the fittest solution. It employs the sine and cosine function in applied mathematical models. Various random and made fit variables are also united into this algorithm to maintain the exploitation and exploration of the search space in many optimization pillars [67]. In this algorithm, the location updating equations are introduced for both stages:

$$\begin{array}{c} P_{i}^{k+1}=P_{i}^{k}+s_{1}\times sin\left(s_{2}\right)\times \left|s_{3}L_{i}^{k}-P_{i}^{k}\right| \end{array}$$ (17)

$$\begin{array}{c} P_{i}^{k+1}=P_{i}^{k}+s_{1}\times cos\left(s_{2}\right)\times \left|s_{3}L_{i}^{k}-P_{ii}^{k}\right| \end{array}$$ (18)

Here, $P_{i}^{k}$ is the location of the present solution in *i*-th aspect at *k*-th iteration, s1/s2/s3 are random numbers, *L*_*i*_ is location of the target point in *i*-th aspect, and || shows the absolute value. These two equations are consolidated as follows:

$$\begin{array}{c} P_{i}^{k+1}=\{\begin{array}{cc} P_{i}^{k}+s_{1}\times sin\left(s_{2}\right)\times \left|s_{3}L_{i}^{k}-P_{i}^{k}\right| & s_{4}<0.5\\ P_{i}^{k}+s_{1}\times cos\left(s_{2}\right)\times \left|s_{3}L_{i}^{k}-P_{ii}^{k}\right| & s_{4}\geq 0.5 \end{array} \end{array}$$ (19)

Here, *s*_4_ ∈ [0, 1] is a random number.

In these equations, there are four central parameters *s*_1_, *s*_2_, *s*_3_, and *s*_4_, where, *s*_1_ manages the subsequent position’s region which is a space among the destination and solution. The parameter *s*_2_ define the movement should be outwards or towards the destination. The parameter *s*_3_ delivers a weight randomly for the target in order to emphasize stochastically (*s*_3_ > 1) or de emphasize (*s*_3_ < 1) the impact of goal in determining the distance. Lastly, *s*_4_ uniformly shifts among the cosine and sine components in Eq 6.

$$\begin{array}{c} s_{1}=a-t\frac{a}{T} \end{array}$$ (20)

The SCA pseudocode is described in the Algorithm 3.

**Algorithm 3** Pseudo code of SCA.

 **Input:** Number of agents (*NA*), Maximum iterations (*Max*_*iter*), Current iteration (*Iter* = 0), Objective function (*f*)

 **Output:** Best agent (*P*_*best*_)

1: **procedure** SCA (*NA*, *Max*_*iter*, *f*, *Iter* = 0)

2:  Initialize the search agents (*P*_*i*_). /* Here, *i* ∈ *NA* */.

3:  Initialize regulating parameters *s*_1_, *s*_2_, *s*_3_, and *s*_4_.

4:  Evaluate each of the search agent (*P*_*i*_) by the objective function *f*.

5:  Identify the current best agent (*P*_*best*_) of population.

6:  **while** *Iter* < *Max*_*iter* **do**

7:   **for** **each** search agent *P*_*i*_ **do**

8:      Update *s*_1_, *s*_2_, *s*_3_, and *s*_4_.

9:      Update the position of search agent *P*_*i*_ using Eq 19.

10:    Evaluate the search agent *P*_*i*_ by the objective function *f*.

11:    Update *P*_*best*_ = *P*_*i*_, **if** *P*_*i*_ is better than the earlier *P*_*best*_).

12:   **end for**

13:   *Iter* = *Iter* + 1

14:  **end while return** *P*_*best*_

15: **end procedure**

#### 4.2.4 Grey-wolf optimizer (GWO)

The GWO algorithm [68] incorporating a grey wolf hunting mechanism into an algorithm that mimicked the leadership structure. This algorithm is used in various areas [69]. In this algorithm, four sorts of grey wolves are utilised to simulate the leadership hierarchy: alpha, beta, delta, and omega. In addition, three primary degrees of hunting are carried out to accomplish optimization: searching for prey, encircling prey, and attacking prey.

The alpha wolf is leader in search space which is responsible making critical and democratic decision in the search space. Some grey wolves assist alpha in decision-making and other pack activities. This wolf is known as beta. These betas are alpha’s subordinate wolves, and they are ranked second in the grey wolf hierarchy. Omega is the Grey wolf’s lowest-ranking member. Omega wolves are required to follow the dictates of other dominant wolves on a regular basis. The wolf that does not belong to an alpha, beta, or omega is known as a delta. They are in charge of omega, but they must also present to alphas and betas.

In order to mathematically model encircling behavior the following equations are proposed:

$$\begin{array}{c} \vec{D}=\left|\vec{C}\vec{X_{p}\left(t\right)}-\vec{X(t)}\right| \end{array}$$ (21)

$$\begin{array}{c} \vec{X(t+1)}=\vec{X_{p}\left(t\right)}-\vec{A}\cdot \vec{D} \end{array}$$ (22)

Here, *t* indicates the current iteration, $\vec{A}$ and $\vec{C}$ are coefficient vectors, $\vec{X_{p}}$ is the position vector of the prey, and $\vec{X}$ indicates the position vector of a grey wolf. The vectors $\vec{A}$ and $\vec{C}$ are calculated as follows:

$$\begin{array}{c} \vec{A}=2\vec{a}\cdot \vec{r_{1}}-\vec{a} \end{array}$$ (23)

$$\begin{array}{c} \vec{C}=2\cdot \vec{r_{2}} \end{array}$$ (24)

Here, components of $\vec{a}$ are linearly decreased from 2 to 0 over the course of iterations and r1, r2 are random vectors in [0, 1]. In order to mathematically recreate grey wolf hunting behaviour, we assume that the alpha (best candidate solution), beta, and delta have a superior understanding of prospective prey locations. As a result, we save the first three best solutions found thus far and require the other search agents (including the omegas) to update their locations in accordance with the best search agents’ placements. In this regard, the following formulas are proposed.

$$\begin{array}{c} \vec{D_{\alpha }}=|\vec{C_{1}}\cdot \vec{X_{\alpha }}-\vec{X}|,\vec{D_{\beta }}=|\vec{C_{2}}\cdot \vec{X_{\beta }}-\vec{X}|,\vec{D_{\delta }}=\left|\vec{C_{3}}\cdot \vec{X_{\delta }}-\vec{X}\right| \end{array}$$ (25)

$$\begin{array}{c} \vec{X_{1}}=\vec{X_{\alpha }}-\vec{A_{1}}\cdot \vec{D_{\alpha }},\vec{X_{2}}=\vec{X_{\beta }}-\vec{A_{2}}\cdot \vec{D_{\beta }},\vec{X_{3}}=\vec{X_{\delta }}-\vec{A_{3}}\cdot \vec{D_{\delta }} \end{array}$$ (26)

$$\begin{array}{c} \vec{X(t+1)}=\frac{\vec{X_{1}}+\vec{X_{2}}+\vec{X_{3}}}{3} \end{array}$$ (27)

The final position is observed to be in a random location within a circle defined by the positions of alpha, beta, and delta in the search space. To put it another way, alpha, beta, and delta estimate the prey’s location, while other wolves update their positions at random around the prey. The pseudo code of the GWO algorithm is given in Algorithm 4.

**Algorithm 4** Pseudo code of GWO.

 **Input:** Number of agents (*NA*), Maximum iterations (*Max*_*iter*), Current iteration (*Iter* = 0), Objective function (*f*)

 **Output:** Best agent (*P*_*α*_)

1:  **procedure** GWO (*NA*, *Max*_*iter*, *f*, *Iter* = 0)

2:  Initialize the agents (*P*), and algorithmic parameters *a*, *A*, and *C*.

3:  Compute the fitness of all agents through function *f*.

4:  Identify the first best (*P*_*α*_), second best (*P*_*β*_), and third best *P*_*δ*_ search agents of population.

5:  **while** *Iter* < *Max*_*iter* **do**

6:   **for** **each** search agent *i* ∈ (*NA*) **do**

7:    Update the position of current search agents (*P*_*i*_) through position update Eq 27.

8:   **end for**

9:   Update *a*, *A*, and *C*.

10:   Compute the fitness of all search agents through function *f*.

11:   Update *P*_*α*_, *P*_*β*_, and *P*_*δ*_.

12:   *Iter* = *Iter* + 1

13:   **end while**

14: **return** *P*_*α*_

15: **end procedure**

## 5 Proposed methodology

The proposed methodology is developed to analyzed the applicability of the meta-heuristics algorithms.

**Algorithm 5** Pseudo code of the proposed Algorithm.

 **Input:** Considered SRGM models for raking, and considered Dataset for evaluation

 **Output:** Report the rank algorithm for considered SRGM models.

1: **procedure** For each ‘*i*^*th*^’ considered model:

 (a) Find the optimal parameter values through statistical learning.

 (b) Compute the Mean Squared Error ($MSE_{sl}^{i}$) between actual and predicted faults.

2:  For each ‘*j*^*th*^’ considered model:

 (a) Initialize the parameter values within their respective ranges.

 (b) Predict the faults at various interval frames of given dataset with initial parameter set.

 (c) Compute the initial sum of squared error (*SSE*^*th*^) between actual and predicted faults.

 (d) For each ‘*j*^*th*^’ Nature-inspired optimization algorithm from set ‘RGA’, ‘GSA’, ‘SCA’, ‘GWO’:

 (e) Return the vector of SRGM parameter values, MSE, and the number of iterations to converge for all the employed algorithm from set ‘RGA’, ‘GSA’, ‘SCA’, ‘GWO’ for ‘*j*^*th*^’ model.

 (f) Rank the employed algorithms based on their number of iteration to converge for the considered ‘*i*^*th*^’ model.

 (g) Return the MSEir1 (i.e., MSE value of rank-1 algorithm), and its iterations values for the considered ‘*i*^*th*^’ model.

3:  For each ‘*i*^*th*^’ considered model

4: (a) Compare $MSE_{sl}^{i}$, and $MSE_{r1}^{i}$ values.

 (b) If $MSE_{r1}^{i}≢MSE_{sl}^{i}$: Report the failure of proposed algorithm.

 (c) Else:

   **return** the rank-1 algorithm for considered SRGM model.

5: **end procedure**

These algorithms are used to estimate the models parameters.

**Fig 1:**
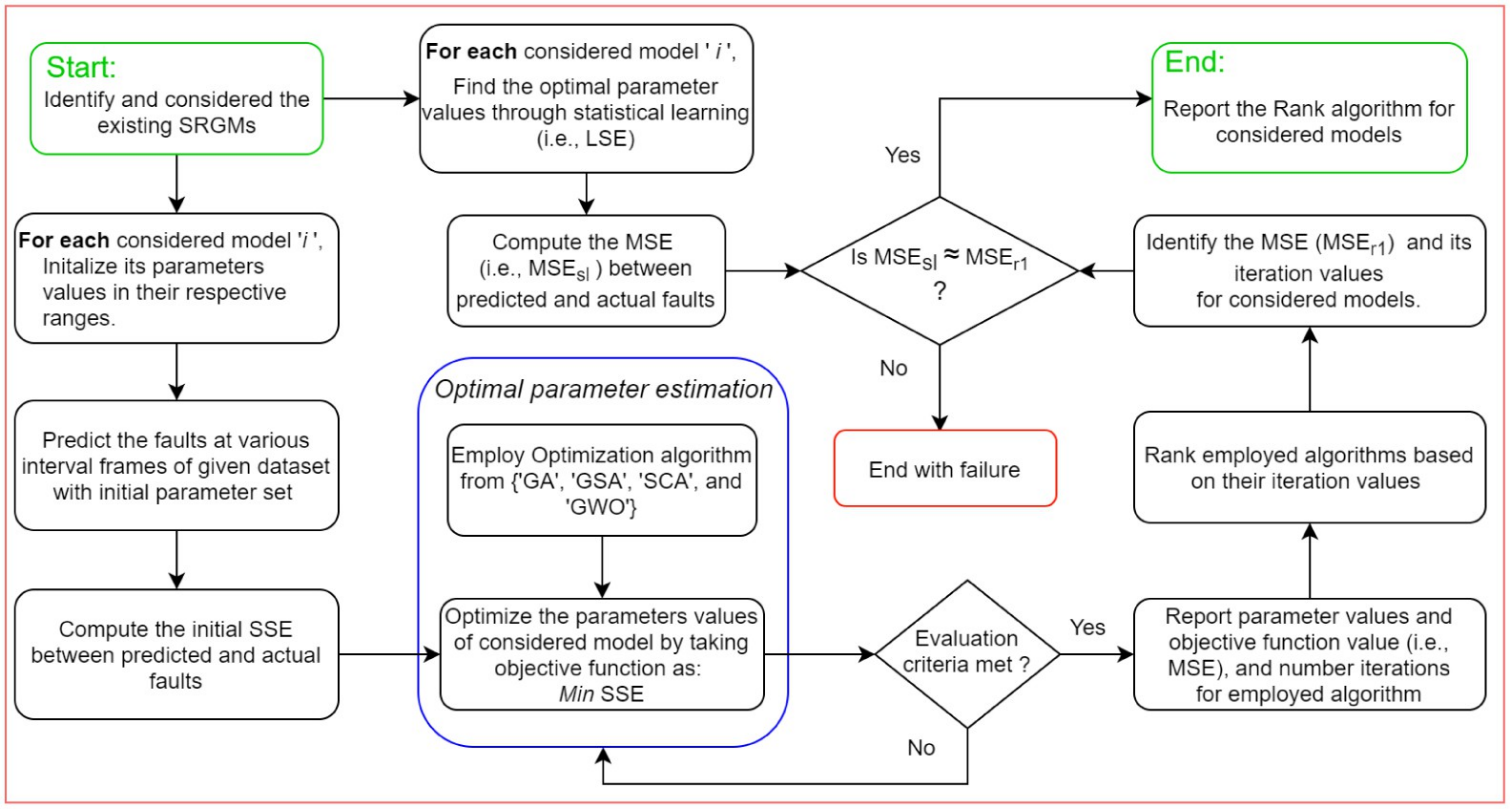
Methodology work-flow diagram.

The process of estimation is distributed into two categories, i.e., traditional methods and meta-heuristics algorithms. Generally, in the past literature, statistical techniques were employed for the parameters estimation of SRGMs. In traditional method LSE and MLE are considered. In recent past various nature-inspired meta-heuristics algorithms like GA, PSO, ACO, GSA, GWO, etc. are used for model parameter estimation. In this study, we consider RGA, GSA, SCA and GWO for models parameter estimation. The complete work-flow of the offered procedure is presented in Fig 1.

Start with SRGM identification, for SRGM selection we consider two, three, four and five parameter well-known SRGMs. For model and algorithm validation we used three real-failure datasets. We see that the parameter values through LSE and meta-heuristic algorithms. The value of parameters are nearly close to LSE values. Based on that parameter values calculate the several comparison criteria for model and algorithm comparison. For the algorithms comparison we evaluate the convergence based on the number of iteration. also perform the *R*^2^ distribution by performing several trails. This proposed method ultimately suggest the best algorithm out of the these competing algorithms based on these convergence and *R*^2^ distribution criteria.

## 6 Experiments and data analysis

This section presents the considered datasets, implementation details, evaluation metrics, and model-wise comparison in detail.

### 6.1 Data description

For this research and comparison, a total of three data sets are employed. Table 2 lists the data sets in detail.

**Table 2:** Single release data sets.

Data-set	Testing Week	Detected faults	Corrected faults	Description
DS-1 [70]	17	144	143	A middle type software project
DS-2 [71]	17	54	54	From software project failure of system 2
DS-3 [72]	14	38	38	Software systems project failure of Real time command and control

The most critical task after model creation is parameter estimate. The popular traditional technique LSE and four meta heuristic techniques RGA, GSA, SCA, and GWO are applied for parameter estimation. As a result, we use three data sets to estimate the SRGMs parameters. The estimated values of the model’s parameters, as well as the goodness of fit, are listed in the Tables 3–6.

**Table 3:** GO model estimated parameter values and goodness of fit.

Data-set	Estimation Technique	Parameters		MSE	PRR	PP
		a	*β*			
DS-1	LSE	22.7600	0.1860	0.8970	0.117	0.152
	RGA	22.7426	0.1863	0.8969	0.117	0.152
	GSA	22.7501	0.1831	0.9124	0.114	0.139
	SCA	22.8136	0.1854	0.8975	0.116	0.151
	GWO	22.7352	0.1865	0.8969	0.117	0.152
DS-2	LSE	31.7170	0.1900	2.2960	0.550	3.188
	RGA	31.6557	0.1906	2.3071	0.551	3.197
	GSA	31.7012	0.1919	2.3139	0.552	3.274
	SCA	31.6249	0.1912	2.3075	0.552	3.213
	GWO	31.6302	0.1905	2.3072	0.551	3.209
DS-3	LSE	136.050	0.1380	33.310	0.477	0.260
	RGA	135.975	0.1383	33.308	0.474	0.260
	GSA	138.331	0.1346	34.561	0.499	0.272
	SCA	136.466	0.1367	33.347	0.496	0.268
	GWO	135.965	0.1382	33.308	0.475	0.260

**Table 4:** ISS model estimated parameter values and goodness of fit.

Data-set	Estimation Technique	Parameters			MSE	PRR	PP
		a	*β*	*ξ*			
DS-1	LSE	22.7600	0.1860	0.0010	0.9790	0.117	0.151
	RGA	22.7427	0.1864	5.7E-16	0.8969	0.117	0.152
	GSA	22.3658	0.2033	0.0745	0.9099	0.117	0.156
	SCA	22.7718	0.1857	0	0.8970	0.116	0.150
	GWO	22.7479	0.1863	1.8E-04	0.8969	0.117	0.152
DS-2	LSE	31.7170	0.1900	1.0E-5	2.3840	0.550	3.188
	RGA	31.6557	0.1906	1.1E-15	2.3959	0.551	3.196
	GSA	31.7751	0.1885	0.0034	2.4036	0.549	3.108
	SCA	31.6356	0.1906	0	2.3962	0.551	3.192
	GWO	31.6552	0.1906	1.1E-05	2.3959	0.551	3.196
DS-3	LSE	136.050	0.1380	0.0001	34.827	0.477	0.260
	RGA	135.974	0.1383	9.4E-16	34.822	0.475	0.260
	GSA	133.837	0.1437	0.0142	36.078	0.455	0.253
	SCA	135.976	0.1382	0	34.823	0.476	0.261
	GWO	135.982	0.1382	0	34.822	0.476	0.260

**Table 5:** PNZ model estimated parameter values and goodness of fit.

Data-set	Estimation Technique	Parameters				MSE	PRR	PP
		a	*β*	*ξ*	*ζ*			
DS-1	LSE	11.6730	1.1480	0.0660	7.7990	0.3740	0.085	0.061
	RGA	11.6396	1.1544	0.0665	7.8830	0.3399	0.087	0.061
	GSA	11.5322	1.3227	0.0673	10.954	0.3803	0.136	0.089
	SCA	11.6022	1.1225	0.0672	7.0940	0.3419	0.068	0.052
	GWO	11.6543	1.1506	0.0663	7.8228	0.3399	0.085	0.060
DS-2	LSE	22.8290	1.0150	0.0170	12.0480	1.4010	0.121	0.186
	RGA	22.5975	1.0579	0.0176	13.4061	1.3102	0.104	0.147
	GSA	22.6790	1.0558	0.0175	12.8558	1.3232	0.117	0.180
	SCA	22.7190	0.9837	0.0173	10.6920	1.3165	0.138	0.237
	GWO	22.6027	1.0564	0.0176	13.3469	1.3102	0.104	0.149
DS-3	LSE	81.5620	0.3370	0.0330	0	9.6480	0.036	0.032
	RGA	81.2388	0.3398	0.0333	0	9.6452	0.035	0.031
	GSA	74.5604	0.4052	0.0404	0.0082	11.196	3.408	1.608
	SCA	80.7660	0.3425	0.0338	0	9.6555	0.035	0.031
	GWO	81.2455	0.3398	0.0333	0.0004	9.6465	0.035	0.031

**Table 6:** PZM model estimated parameter values and goodness of fit.

Data-set	Estimation Technique	Parameters					MSE	PRR	PP
		a	*β*	*ξ*	*ζ*	*k*			
DS-1	LSE	73.2800	1.1760	0.0140	7.9000	0.2880	0.4320	0.090	0.064
	RGA	71.1331	1.1737	0.0144	7.8639	0.7333	0.4323	0.089	0.063
	GSA	54.1117	1.2550	0.0203	11.991	0.3193	0.5287	0.336	0.145
	SCA	91.9110	1.1423	0.0105	7.3797	0	0.4287	0.076	0.056
	GWO	116.251	1.1105	0.0078	6.9368	0.0021	0.4263	0.067	0.052
DS-2	LSE	30.0290	0.2160	10.035	0	1.3330	2.3600	0.329	0.771
	RGA	28.9486	0.2168	9.6433	2.7E-17	2.3520	2.3713	0.328	0.771
	GSA	27.3166	0.2121	10.449	3.6E-04	3.9834	2.3814	0.359	0.940
	SCA	31.2919	0.2174	10.082	0	0	2.3715	0.319	0.716
	GWO	27.8623	0.2168	9.2906	0.0002	3.4386	2.3714	0.328	0.771
DS-3	LSE	145.209	1.2240	0.0660	1.111	16.699	6.3900	0.020	0.020
	RGA	143.7669	1.2144	0.0663	1.0789	17.899	6.3764	0.021	0.020
	GSA	143.8802	1.2083	0.0665	1.5422	19.011	7.7855	0.042	0.036
	SCA	158.6906	1.3535	0.0703	1.0857	0	6.7478	0.020	0.021
	GWO	141.7625	1.1982	0.0661	1.0377	20	6.3542	0.021	0.020

### 6.2 Model evaluation criteria

The Mean of Squared Errors (MSE), Predictive power (PP) and Predictive ratio risk (PRR), on the data set are used to generate the evaluation fitness value of the set of parameters. Three criteria for comparison are utilized to assess SRGM’s descriptive enactment. The following are the criteria:

1. **MSE:** MSE is a metric that is used to compare prediction models quantitatively. The distinction between the true and anticipated value is computed using MSE. It is illustrated as follows:
  $$\begin{array}{c} \text{MSE}=\frac{1}{n}\sum_{i=1}^{n}{\left[\vartheta \left(t_{i}\right)-y_{i}\right]}^{2}, \end{array}$$ (28)
  Here, *ϑ*_*i*_ is the monitored numeral of noticed defects by time *t*_*i*_ and MVF at time *t*_*i*_ is represented as *ϑ*(*t*_*i*_). It is desirable if the value of MSE is smaller by the provided model.
2. **Predictive ratio risk (PRR):** It compares the model’s estimated distance from the actual data to the model’s estimated distance from the fitted curve. In this case, a lower PRR value indicates that the fitted curve is better suited to the failure data.
  $$\begin{array}{c} PRR=\sum_{i=1}^{n}{\left(\frac{u\left(t_{i}\right)-y_{i}}{u(t_{i})}\right)}^{2}, \end{array}$$ (29)
3. **Predictive power (PP):** The difference between the model estimate and the actual data is calculated using predictive power.
  $$\begin{array}{c} PP=\sum_{i=1}^{n}{\left(\frac{u\left(t_{i}\right)-y_{i}}{y_{i}}\right)}^{2}, \end{array}$$ (30)
  We see the performance, i.e., MSE, PRR and PP of LSE and four meta heuristic techniques on SRGMs from the below tables.

### 6.3 Model wise comparison

Because the results of a single run may be incorrect due to meta-heuristics’ stochastic character, all of the algorithms are run 30 times, with the best results gathered and published in Tables 3–6 for GO, ISS, PNZ and PZM models, respectively.

**Fig 2:**
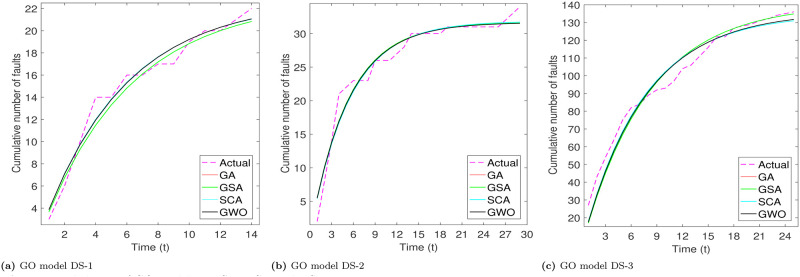
Fitted shapes of GO model on DS-1, DS-2 and DS-3. (a) GO model DS-1, (b) GO model DS-2, (c) GO model DS-3.

**Fig 3:**
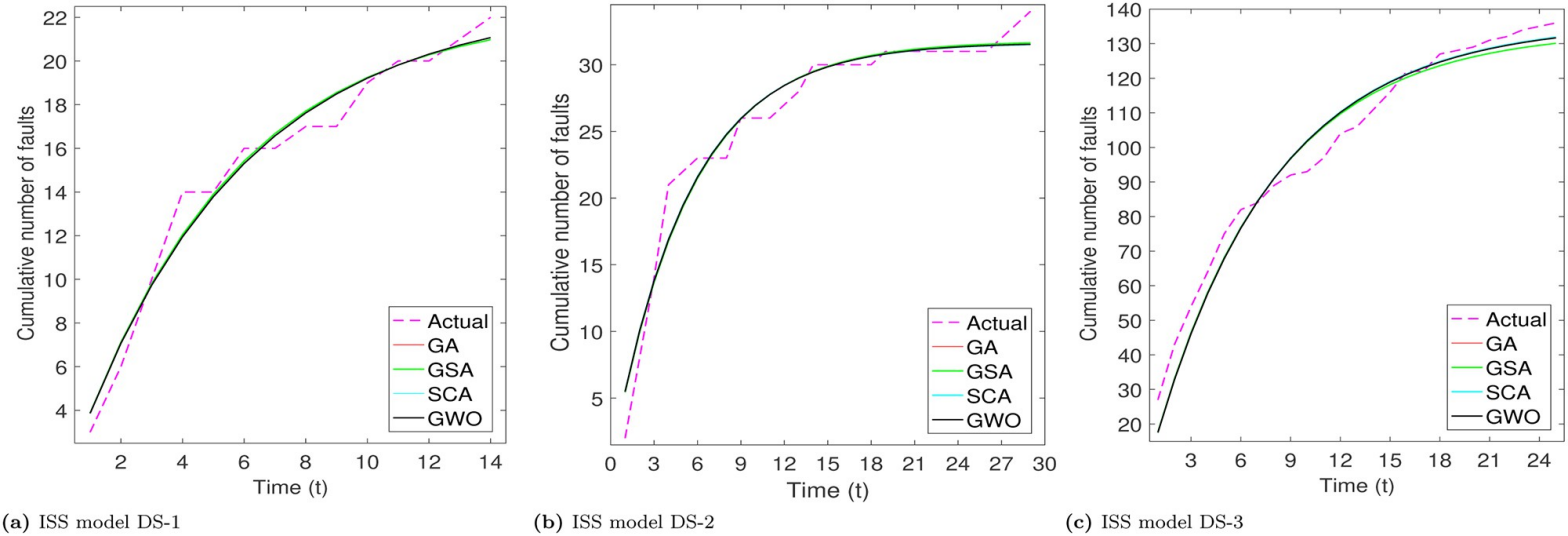
Fitted shapes of ISS model on DS-1, DS-2 and DS-3. (a) ISS model DS-1, (b) ISS model DS-2, (c) ISS model DS-3.

**Fig 4:**
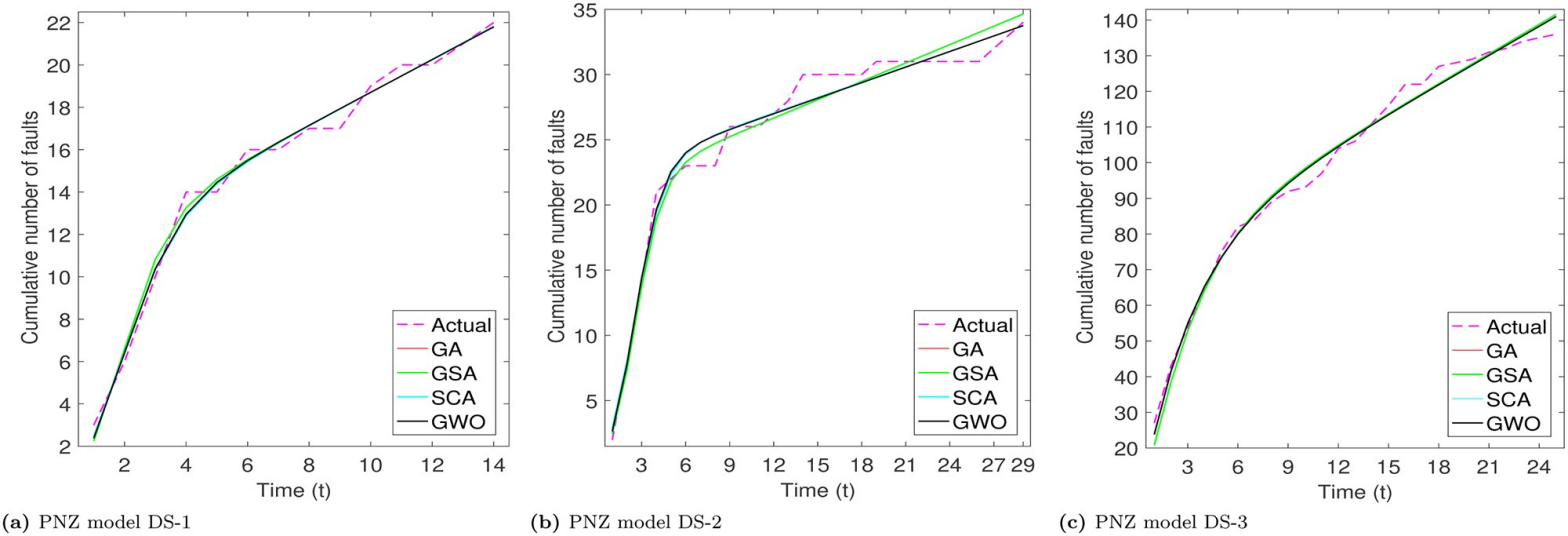
Fitted shapes of PNZ model on DS-1, DS-2 and DS-3. (a) PNZ model DS-1, (b) PNZ model DS-2, (c) PNZ model DS-3.

**Fig 5:**
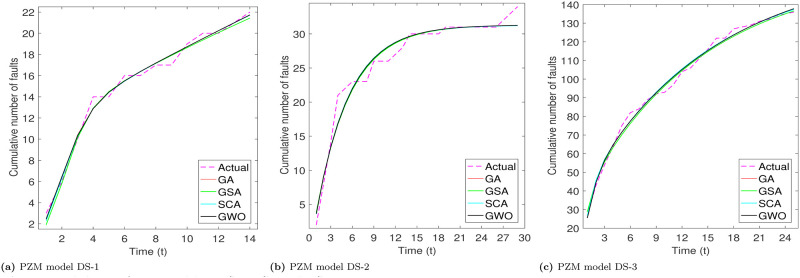
Fitted shapes of PZM model on DS-1, DS-2 and DS-3. (a) PZM model DS-1, (b) PZM model DS-2, (c) PZM model DS-3.

From Tables 3–6, we can see that PZM model perform better for DS-1 and DS-3, while for DS-3, PNZ perform better. The another thing we see that all considered meta-heuristic algorithms also perform better or approx to the LSE method. Based the analysis of goodness of fit table we analyze that RGA and GWO produce almost close values. After the models parameter estimation, we plot the actual and predicted cumulative number of faults. All the considered models are plotted below for all the three datasets in Figs 2–5.

### 6.4 Meta-heuristic algorithm evaluation criteria

#### 6.4.1 Convergence wise comparison

**Fig 6:**
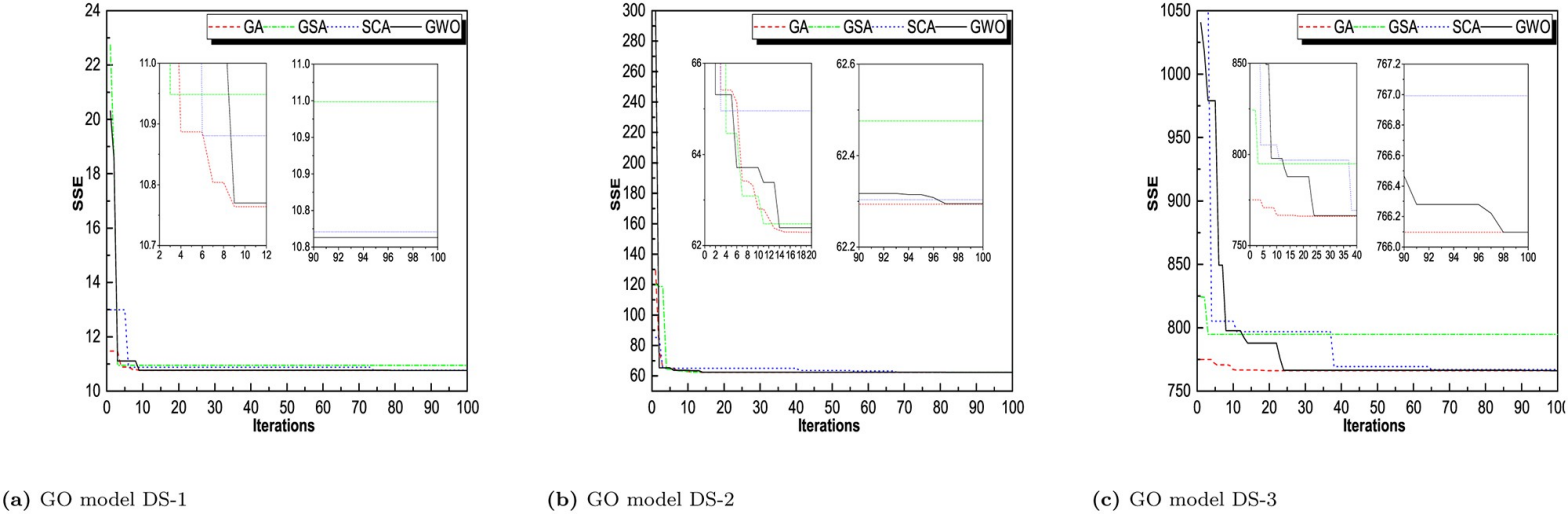
Convergence curve of RGA, GSA, SCA, and GWO for GO model on DS-1, DS-2, and DS-3. (a) GO model on DS-1, (b) GO model DS-2, (c) GO model DS-3.

**Fig 7:**
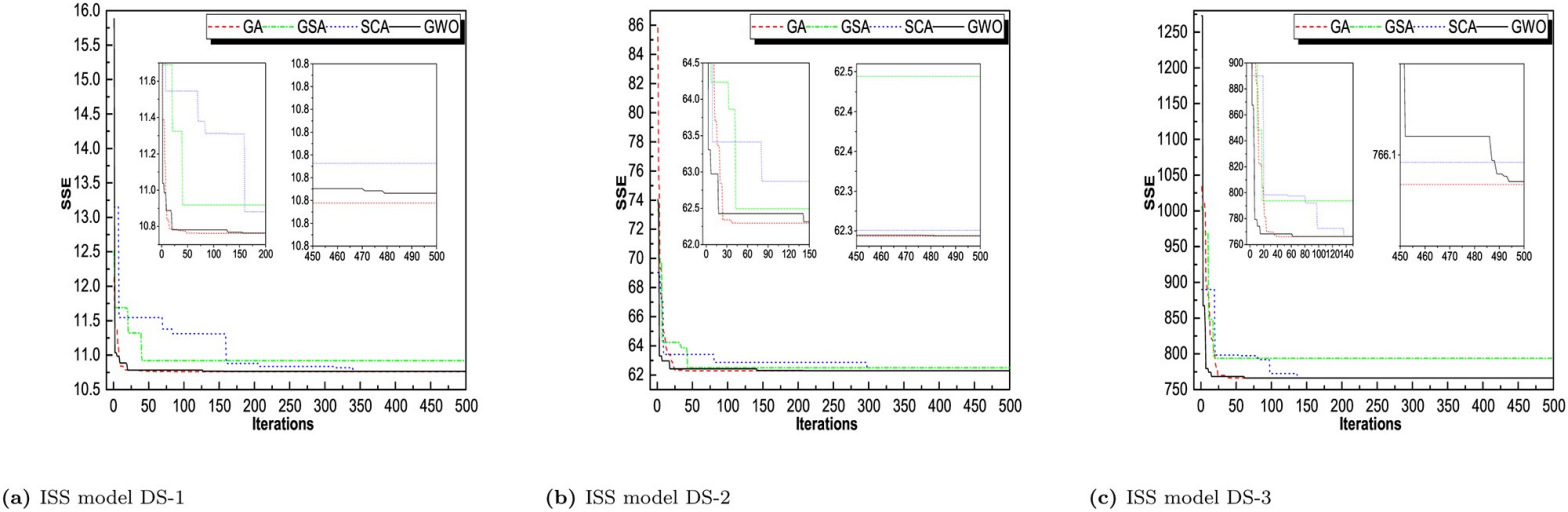
Convergence curve of RGA, GSA, SCA, and GWO for ISS model on DS-1, DS-2, and DS-3. (a) ISS model DS-1, (b) ISS model DS-2, (c) ISS model DS-3.

**Fig 8:**
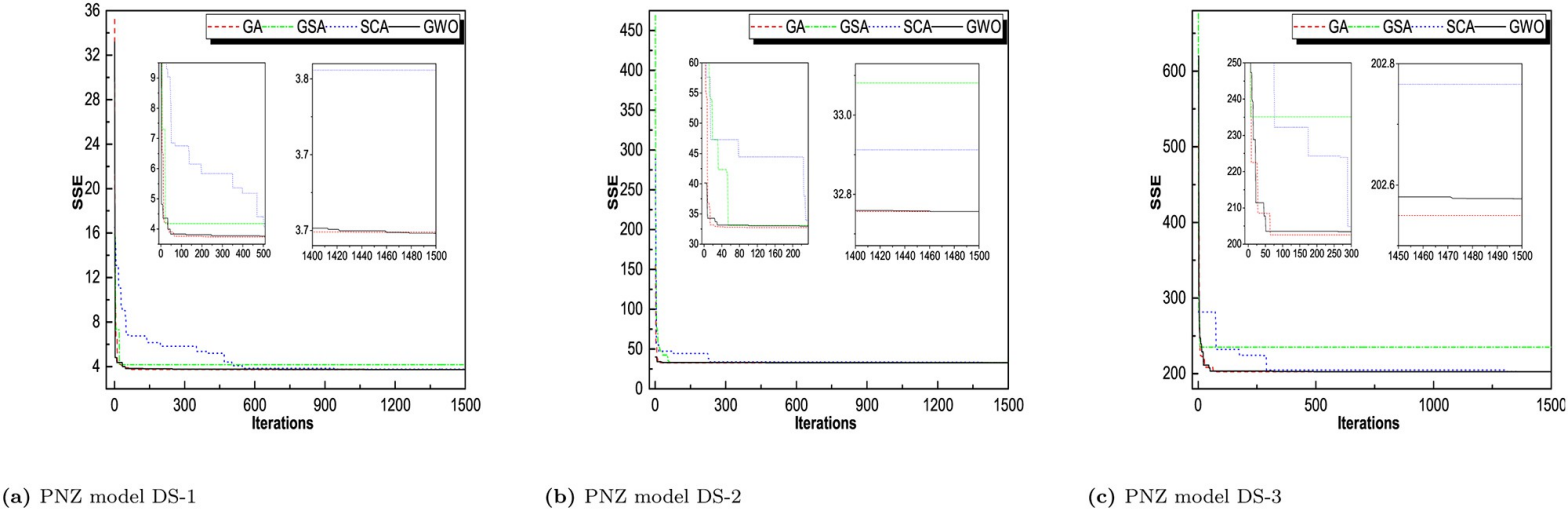
Convergence curve of RGA, GSA, SCA, and GWO for PNZ model on DS-1, DS-2, and DS-3. (a) PNZ model DS-1, (b) PNZ model DS-2, (c) PNZ model DS-3.

**Fig 9:**
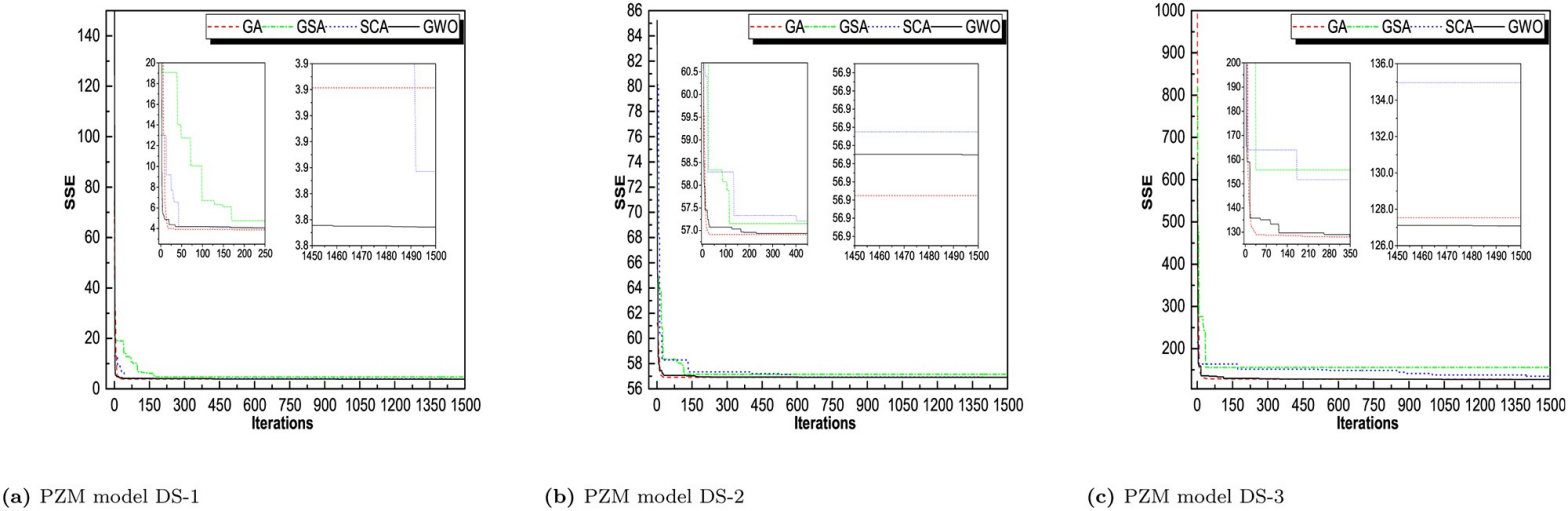
Convergence curve of RGA, GSA, SCA, and GWO for PNZ model on DS-1, DS-2, and DS-3. (a) PNZ model DS-1, (b) PNZ model DS-2, (c) PNZ model DS-3.

Throughout iterations, the fitness of tracking agents degrades as shown in the observed patterns in Figs 6–9. This demonstrates that the algorithms under consideration are capable of improving the wellness of initial random solutions for a provided optimization situation in the long run. As shown in these diagrams, the search agents of the algorithms prefer to investigate the advantageous territories of the tracking space before settling on the optimal one. The algorithms’ convergence manners were examined and validated. That can be derived indirectly from the average fitness and trajectory, which are illustrated in these pictures as the convergence curves of RGA and GWO. The most reasonable explanation found so far during optimization is depicted in these diagrams. The downward tendency can be seen in the convergence curves of RGA and GWO for all of the SRGMs and datasets studied. For GO model, DS-1, RGA and GWO converges in less than 10 iterations. For DS-2, RGA, GWO, and GSA converges less than 20 iterations. For DS-3, RGA take 10, GWO take 25 and SCA takes around 40 iterations to converge the optimal solution. Similarly, for rest of the models we see that RGA converges faster and GWO also converges approximate iterations. The convergence plot for all the models for all the datasets are presented in Figs 6–9.

For comparison between RGA and GWO, we concluded that RGA performs better and converges in smaller no. of iterations for most of the datasets.

#### 6.4.2 Comparison based on *R*^2^ distribution

**Fig 10:**
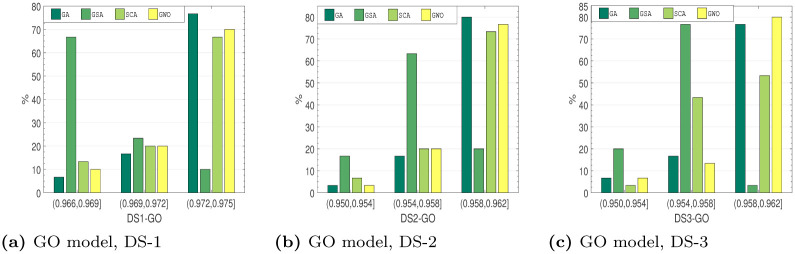
*R*^2^ value distribution of GO model on DS-1, DS-2 and DS-3. (a) ISS model, DS-1, (b) ISS model, DS-2, (c) ISS model, DS-3.

**Fig 11:**
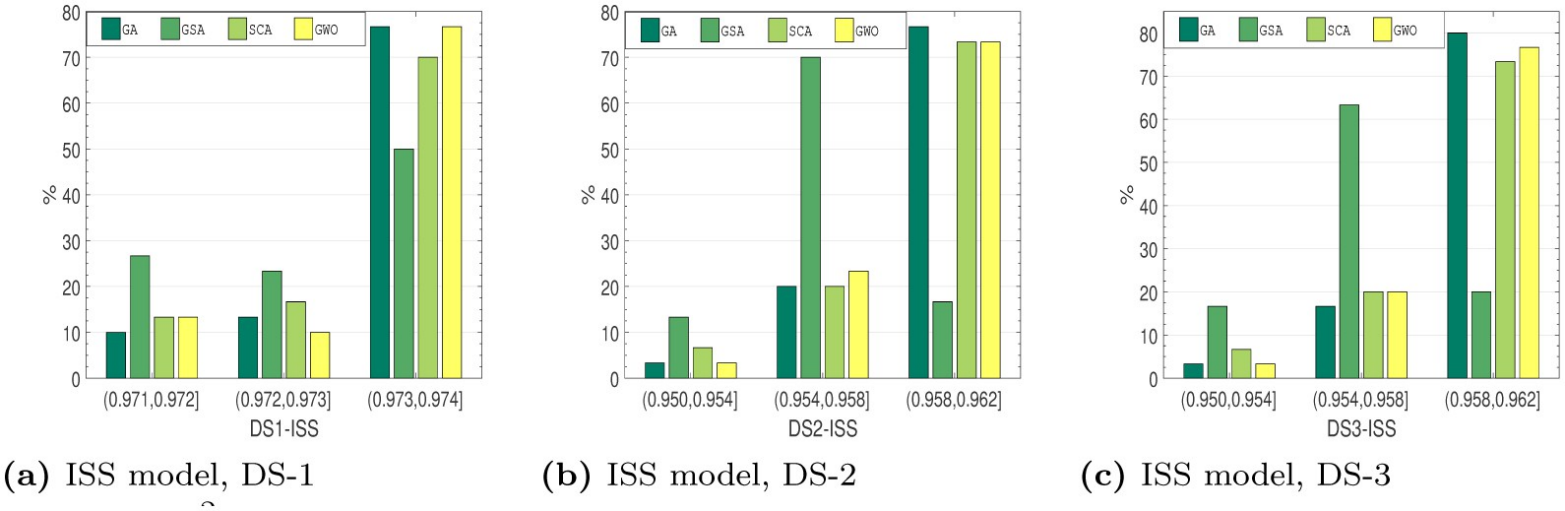
*R*^2^ value distribution of ISS model on DS-1, DS-2 and DS-3. (a) ISS model, DS-1, (b) ISS model, DS-2, (c) ISS model, DS-3.

**Fig 12:**
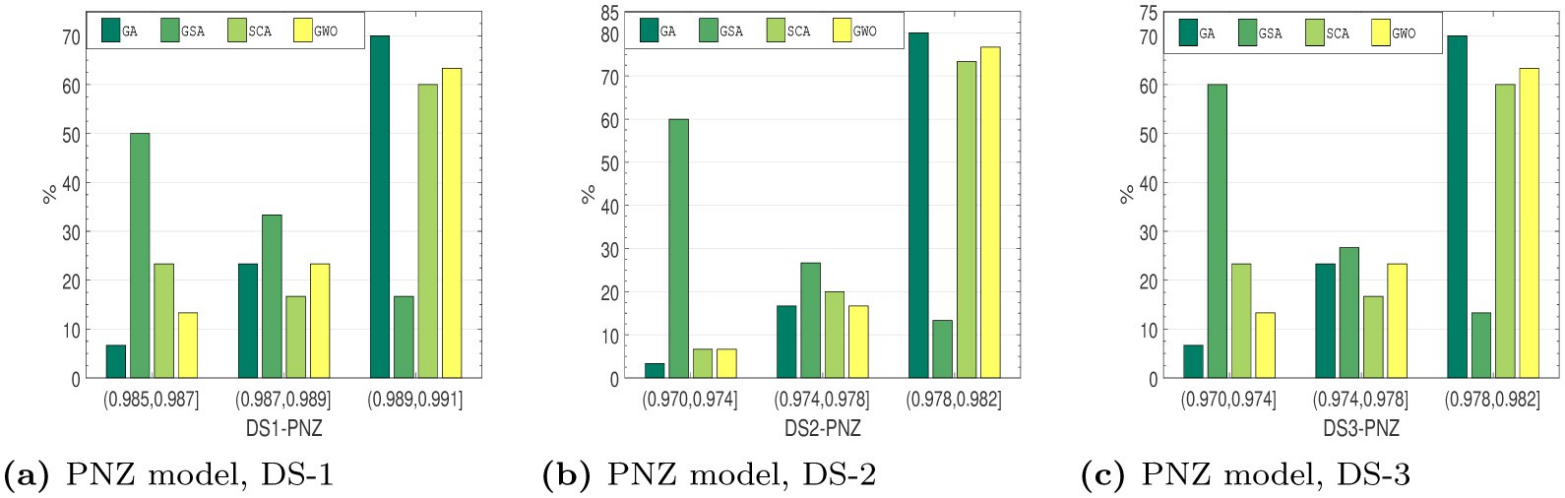
*R*^2^ value distribution of PNZ model on DS-1, DS-2 and DS-3. (a) PNZ model, DS-1, (b) PNZ model, DS-2, (c) PNZ model, DS-3.

**Fig 13:**
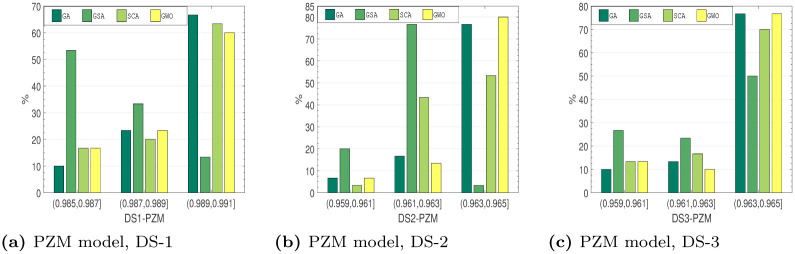
*R*^2^ value distribution of PZM on DS-1, DS-2 and DS-3. (a) PZM model, DS-1, (b) PZM model, DS-2, (c) PZM model, DS-3.

*Distribution characteristics analysis*. Several approaches to assess the outstanding performance of the algorithms have been employed. Here, this work examines the distribution features of the employed method on the testing collection. These optimization outcomes of the recommended method at all run are not certainly the equivalent. To assess the optimization strength and the reliability, the stated method runs 30 times on all testing set. Figs 10–13, shows the observe *R*^2^ value distributions of all four soft-computing techniques on the testing set of DS1-DS3. The cumulative value of *R*^2^ is larger than 0.95 for all the SRGMs for all the datasets. From Figs 10–13, we can see that the RGA and GWO perform better in third interval, i.e., highest *R*^2^ value interval. Although the GSA and SCA is quite reasonable, the absolute deviation only 0.009, *R*^2^ value distributions performance of RGA and GWO is considerably greater than the other two examined algorithms.

## 7 Conclusion and future work

This work analyzes the various meta-heuristic algorithms used in estimating the parameters of SRGMs. This is the first attempt to use several meta-heuristic approaches to estimate the parameters of numerous SRGMs. The considered model is better fit when the SRGM parameters are optimized accurately. The major inference of this work are given as follows: The work does not require any hypotheses/constrains for the software failure data for parameter estimation and instead relies solely on the data’s attributes, indicating that its implementation is simple. The proposed approach is to compare the meta-heuristic approaches for parameter estimation by various criteria. The experimental results show that the performance of RGA and GWO is better on a variety of real-world failure data, and they suggest RGA has excellent parameter estimation potential.

In the parameter estimation, the traditional state-art-of-the-methods fail to find feasible solutions on specific SRGMs or datasets, however the suggested meta-heuristic algorithms performs far superior does in this context. The suggested algorithm’s findings are comparable to traditional methods for the four models, namely, Goel-Okumoto, Inflection S-Shape model, PNZ, and PZM model. When computed the optimized value of parameters through meta heuristic methods, the considered approach produces significantly superior results in some cases. LSE method’s outcomes are significantly approx to RGA, GSA, SCA and GWO. RGA could locate the optimal solution more correctly and faster than GWO and other approaches. In this paper, comparison of various estimation technique and selection of best technique for parameter estimation is done.

In future work we typically utilized approaches, such as MLE: (1) the wavelet shrinkage estimation allows us to bring out the time-series analysis with accuracy and high-speed prerequisites, and (2) The wavelet shrinkage estimation is ordered into a non-parametric estimation without setting a parametric form of the software intensity function.
